# Functional Characterization of Rare Genetic Variants in the N-Terminus of Complement Factor H in aHUS, C3G, and AMD

**DOI:** 10.3389/fimmu.2020.602284

**Published:** 2021-01-14

**Authors:** Edwin K. S. Wong, Thomas M. Hallam, Vicky Brocklebank, Patrick R. Walsh, Kate Smith-Jackson, Victoria G. Shuttleworth, Thomas E. Cox, Holly E. Anderson, Paul Nigel Barlow, Kevin James Marchbank, Claire L. Harris, David Kavanagh

**Affiliations:** ^1^ Complement Therapeutics Research Group, Translational and Clinical Research Institute, Newcastle University, Newcastle upon Tyne, United Kingdom; ^2^ National Renal Complement Therapeutics Centre, Royal Victoria Infirmary, Newcastle upon Tyne, United Kingdom; ^3^ School of Chemistry, Joseph Black Building, University of Edinburgh, David Brewster Road, Edinburgh, United Kingdom; ^4^ NIHR Newcastle Biomedical Research Centre, Biomedical Research Building, Campus for Ageing and Vitality, Newcastle upon Tyne, United Kingdom

**Keywords:** complement factor H, age-related macular degeneration, aHUS, C3G, MPGN

## Abstract

Membranoproliferative glomerulonephritis (MPGN), C3 glomerulopathy (C3G), atypical haemolytic uraemic syndrome (aHUS) and age-related macular degeneration (AMD) have all been strongly linked with dysfunction of the alternative pathway (AP) of complement. A significant proportion of individuals with MPGN, C3G, aHUS and AMD carry rare genetic variants in the *CFH* gene that cause functional or quantitative deficiencies in the factor H (FH) protein, an important regulator of the AP. *In silico* analysis of the deleteriousness of rare genetic variants in *CFH* is not reliable and careful biochemical assessment remains the gold standard. Six N-terminal variants of uncertain significance in *CFH* were identified in patients with these diseases of the AP and selected for analysis. The variants were produced in *Pichia Pastoris* in the setting of FH CCPs 1–4, purified by nickel affinity chromatography and size exclusion and characterized by surface plasmon resonance and haemolytic assays as well as by cofactor assays in the fluid phase. A single variant, Q81P demonstrated a profound loss of binding to C3b with consequent loss of cofactor and decay accelerating activity. A further 2 variants, G69E and D130N, demonstrated only subtle defects which could conceivably over time lead to disease progression of more chronic AP diseases such as C3G and AMD. In the variants S159N, A161S, and M162V any functional defect was below the capacity of the experimental assays to reliably detect. This study further underlines the importance of careful biochemical assessment when assigning functional consequences to rare genetic variants that may alter clinical decisions for patients.

## Introduction

Atypical haemolytic uraemic syndrome (aHUS) (1), membranoproliferative glomerulonephritis (MPGN), C3 glomerulopathy (C3G) (2) and age-related macular degeneration (AMD) (3) are all diseases that have been associated with dysregulation of the alternative pathway (AP) of complement.

The hallmark pathological lesion in aHUS is thrombotic microangiopathy within the kidney, characterized by the clinical features of microangiopathic haemolytic anaemia, thrombocytopenia and acute kidney failure (1). Conversely, MPGN and C3G are a group of conditions in which deposition of complement activation products within the glomerulus occurs, resulting in nephrotic/nephritic syndrome and chronic progressive renal failure (2). Specifically, MPGN is a pattern of glomerular injury, involving thickening of the glomerular capillary wall and an increase in mesangial components. C3G is the current preferred term when the glomerular staining is predominantly C3 positive by immunofluorescence. Dense deposit disease is a special sub-type of C3G, characterized by prominent intramembranous laminar deposits that are visible on electron microscopy, associated historically with MPGN (2).

AMD is the leading cause of irreversible vision loss in elderly Caucasian populations. It is characterized by lipoprotein-rich drusen deposits that form in the subretinal space and the recruitment of immune cells, such as microglia and macrophages (4, 5). Degeneration and geographic atrophy of the retinal pigment epithelium and photoreceptors occur in the macula of the retina in advanced dry AMD, while advanced wet AMD involves angiogenesis in the choroid and subsequent choroidal neovascularization (6).

Genetic studies have linked common (7–11) and rare genetic variants (12–17) in the complement factor H gene (*CFH*) to all of these conditions. Complement factor H (FH) is an abundant fluid phase regulator of the AP, and functions as a cofactor for factor I (FI) in the cleavage of the activated central molecule of the pathway, C3b (18, 19). Screening for rare genetic variants in *CFH* has been established in clinical practice to determine a possible genetic cause of disease. Knowledge of *CFH* variants and their functional significance plays an important role in understanding prognosis (20) and determining the risk of disease recurrence after renal transplantation (21) in aHUS. Similar genotype-phenotype correlations have not yet been established in MPGN/C3G. In all of these diseases, common risk haplotypes also associate with pathology and play a role in genetic susceptibility (7–11).

The functional consequences of genetic variants identified in individual patients needs to be carefully considered as they could influence the approach to clinical management of the condition. Because each genetic variant is rare, there may not be any literature reports on its functional impact. There is thus a good case for a robust and consistent procedure to be established for assessing the likelihood that a rare variant in *CFH* is functionally detrimental and hence could be contributing to disease. Functional testing although time-consuming remains the gold standard for attributing significance to a rare genetic variant and has been shown to be contradictory to *in silico* analysis of variants in *CFH* (22).Most rare *CFH* genetic variants in aHUS reported to date have been found to code for amino acids in the C-terminal recognition domain, comprising complement control proteins (CCPs) 19–20 (23). Many variants have been extensively studied in the laboratory and most demonstrate functional significance (14, 24). Variants have also been reported in the N-terminal regulatory domain, comprising CCPs 1–4 and there is significant enrichment of N - terminal variants in AMD (25). To date, there have been functional studies of 10 variants in CFH CCPs 1-4, namely; R53C (16, 22), R53H (15, 26, 27), R53P (22), R53S (22), S58A (22), I62V (15, 27, 28), R78G (15), R83S (13), D90G (16) and Del K224 (29), and in many cases there was a severe loss of function.

The objective of this study was to expand this knowledge pool by testing the functional significance of other variants of uncertain significance identified in the N-terminal region of FH in patients with aHUS, MPGN, C3G, and AMD.

## Materials and Methods

### Selection of N-Terminal *CFH* Variants for Functional Study

At the time of study inception, the literature was reviewed for N-terminal rare genetic variants described in the complement mediated diseases: MPGN/C3G; aHUS, and AMD. Six N-terminal heterozygous variants known to result in normal serum FH levels and for which no prior functional data were available were selected as follows: G69E (17, 25), Q81P (30), D130N (25, 31), S159N (25, 32, 33), A161S (25, 30, 31, 33, 34) and M162V (35).

### Production and Purification of Proteins

Clones of *Pichia pastoris* strain KM71H producing wild-type (WT) and mutant (G69E, Q81P, D130N, S159N, A161S, and M162V) protein in the setting of CCPs1–4 were generated as described previously (15). In brief, each point mutation was generated in a pPICZαB (Invitrogen) vector containing residues 19–263 of FH (which encodes CCPs 1–4 of mature FH; residues 1–18 are the mammalian signal peptide), with a C-terminal 6x His tag and an N-terminal myc tag (EQKLISEEDL) using QuikChange site-directed mutagenesis kit (Stratagene). Fidelity was confirmed by bi-directional Sanger sequencing. KM71H cells were transformed using electroporation, selected for by zeocin, and screened for protein expression.

Protein expression was carried out in a 3L BioFlo 115 Biofermenter (New Brunswick). A starter culture was transferred into 1L of basal fermentor salts (0.095%) (w/v) calcium sulphate, 1.82% (w/v) potassium sulphate, 1.5% (w/v) magnesium sulphate heptahydrate, 0.42% (w/v) potassium hydroxide, 2.7% (v/v) phosphoric acid and 2.5% (v/v) glycerol) enriched with 1% (w/v) casein amino acids, 0.5% (w/v) PTM1 salts and 0.5% (v/v) antifoam A (Sigma). After the initial batch fed glycerol was exhausted, software-controlled glycerol feeds were maintained for 24 h at 30°C. The cells were allowed to starve for 4 h before recombinant expression was induced with 0.75% methanol containing 0.5% (w/v) PTM1 salts. After three days at 15°C with software-controlled methanol feeds, cells were spun out and the supernatant was removed, filtered and its pH adjusted to 7.4.

The supernatant was then applied to a 5ml His-Trap FF column (GE-healthcare) at 4°C and the protein eluted with 500 mM imidazole followed by size exclusion chromatography using a HiLoad^®^ 16/600 Superdex^®^ 200 pg column (GE Healthcare) (**Supplementary Figure 1**). Protein concentrations were calculated using absorbance at 280 nm and a calculated extinction coefficient (478,70M cm^−1^).

### Binding Affinity for C3b by Surface Plasmon Resonance

The binding affinities of the WT and mutant FH1-4 proteins to C3b were monitored by SPR using a Biacore X100 instrument (GE Healthcare) (14). A total of 300 resonance units of human C3b (CompTech) were immobilized on a Biacore series S-CM5 sensor chip (GE Healthcare) using standard amine coupling. The reference surface of the chip was prepared by performing a mock coupling in the absence of any protein. Experiments were performed at 25°C and 30 μl/min flow rate. Duplicate injections (concentrations up to 40 μM) were performed in 10 mM HEPES-buffered saline, 3 mM EDTA, 0.05% (v/v) surfactant p20 (GE Healthcare). A contact time of 45s was used (sufficient for achieving steady state conditions for most samples) followed by a dissociation period of 90 s. Chips were regenerated between sample injections, with one 45 s injections of 1 M NaCl, pH 7.0. Data were processed using the BIAevaluation 4.1 software (GE Healthcare). Data from the reference cell and a blank (buffer) injection were subtracted and dissociation constants calculated using a steady-state affinity model from the background-subtracted traces.

### Measurement of Decay Acceleration Activity by Surface Plasmon Resonance

Decay accelerating activity (DAA) was measured in real-time using a Biacore X100 instrument as described previously (15). Briefly, 300 resonance units of C3b were immobilized using standard amine coupling to the CM5 sensor chip. Subsequently, a mixture of FB (250 nM) and FD (30 nM) was flowed (10 μl/min) over the surface for 120s to form the AP convertase.

The convertase was allowed to decay naturally for 210s and then FH1-4 (WT or variants at 166nM) [in running buffer, HEPES-buffered saline containing 0.5% (v/v) surfactant P20 and 1 mM MgCl_2_, Temp 25°C] was flowed across the surface for 60s, and convertase decay was visualized in real time. Between injections, surfaces were regenerated using a 45s injection of 1μM purified FH (CompTech) followed by a 45s injection of 1M NaCl, pH7.0. Data were evaluated using BIAevaluation 4.1 (GE Healthcare).

### Cofactor Assay in the Fluid Phase

*In vitro* fluid phase assays were used to measure cofactor activity (CA) for FI-mediated proteolytic cleavage of C3b (14). FI and C3b were purchased from CompTech. An aliquot of 3μL of C3b (5.68 μM), 4.5 μL of FI (0.114 µM) and 5 µL of FH1-4 (0.75 μM) were made up to a final volume of 15μL in phosphate buffered saline (PBS). For the negative control 5 μL of PBS was used. The mixture was incubated at 37°C for 15 min, and the reaction stopped by the addition of 2x Laemmli reducing buffer to a final volume of 30 μL and heated to 95°C for 5 min. The products of the reaction were then separated by SDS-PAGE and revealed using Coomassie Blue staining.

### Measuring Decay Acceleration on Sheep Erythrocytes

C3b-coated sheep erythrocytes (EA-C3b) were prepared as described previously (13). Cells were resuspended to 2% (v/v) in AP buffer (5 mM sodium barbitone, pH 7.4, 150 mM NaCl, 7 mM MgCl_2_, 10 mM EGTA). The AP convertase was formed on the cell surface by incubating 50μL of cells with an equal volume of AP buffer containing FB (40μg/ml) and FD (0.4 μg/ml; CompTech) at 37°C for 15 min. Cells (100 μl) were incubated with 50 μl of a concentration range of FH1–4 (WT or variants) in PBS/20 mM EDTA for 15 min. Lysis was developed by adding 50 μl of 4% (v/v) normal human serum depleted of FB and FH (NHSΔBΔH) (13) in PBS/20 mM EDTA and incubating at 37°C for 60 min. To determine the amount of lysis, cells were pelleted by centrifugation, and hemoglobin release was measured at 410 nm (A410). Controls included 0% lysis (buffer only) and 100% lysis (0.1% (v/v) Nonidet P-40), Percentage of inhibition from lysis was calculated by the formula (A_410_[buffer only]-A_410_[FH])/A_410_[buffer only]*100%.

### Measuring Cofactor Activity on Sheep Erythrocytes

To test CA (13), washed EA-C3b cells were resuspended to 2% (v/v) in AP buffer and incubated with an equal volume of a range of concentrations of FH1–4 (WT and variants) and 2.5 μg/ml FI (CompTech) for 15 min at 25°C. After three washes in AP buffer, a 50 µl aliquot of cells (2%) was mixed with 50 μl AP buffer containing FB (40 μg/ml) and FD (0.4 μg/ml) and then incubated for 15 min at 25°C to form AP convertase on the remaining C3b. Lysis was developed by adding 50 μl of 4% (v/v) NHSΔBΔH in PBS/20 mM EDTA and incubating at 37°C for 10 min. Percentage of inhibition from lysis was calculated by the formula (A_410_[buffer only]-A_410_[FH])/A_410_[buffer only]*100%.

## Results

### Expression of Recombinant FH1-4 Variants

**Figure 1:**
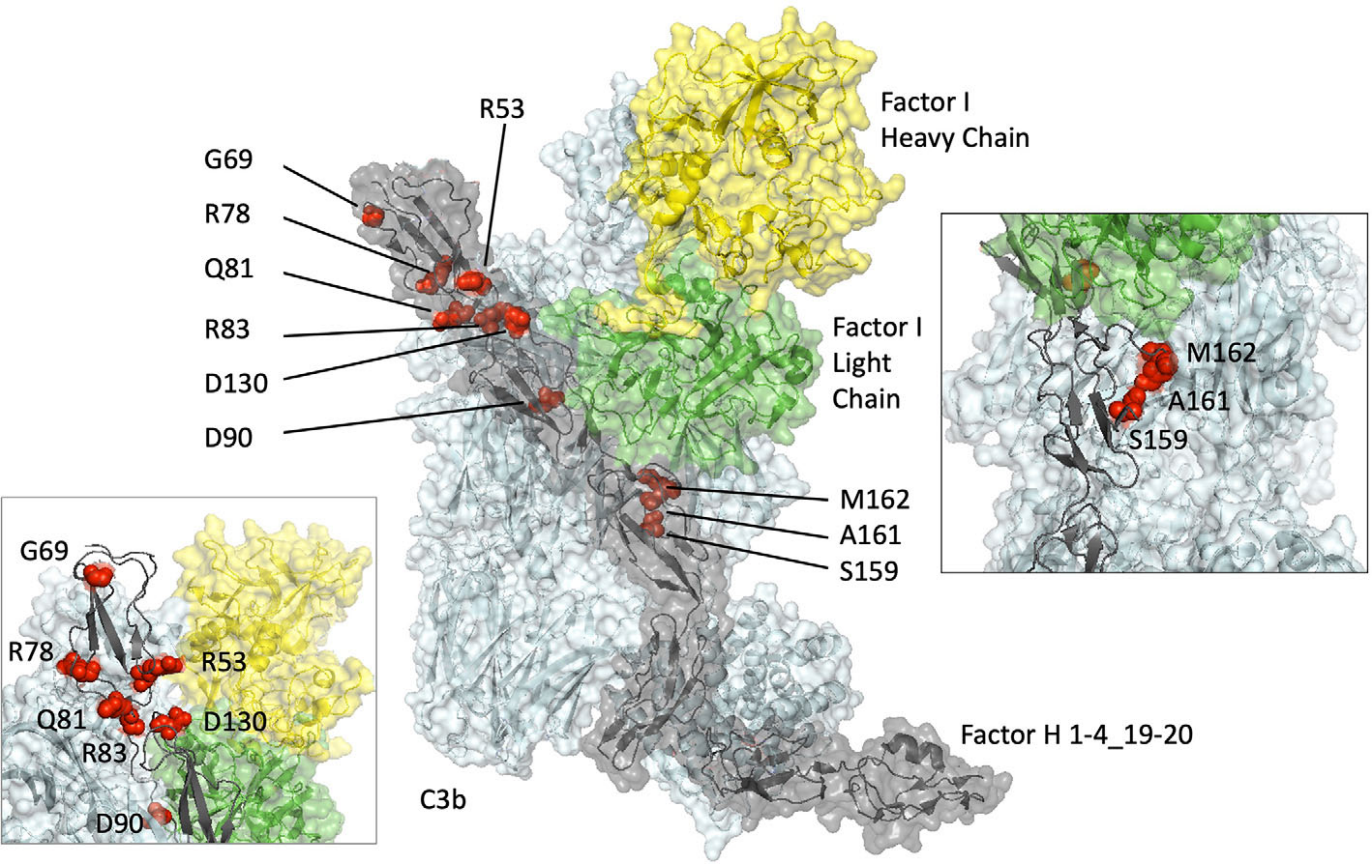
Structural modelling of rare genetic variants in an x-ray–derived co-crystal structure of Factor H CCP1-4_19-20/C3b/Factor I displayed using PyMOL (V2.0.6, Schrödinger, LLC). The amino acids altered by the rare genetic variations analyzed in this study (G69, Q81, D130, S159, A161, M162) and previously (R53 (15, 16, 22), R78 (15), R83 (13), D90 (16)) (red spheres) are shown within a co-crystal structure of Factor I (heavy chain: yellow, light chain: green), Factor H (grey) and C3b (cyan). (Protein Database ID code 5O32) (36).

The rare genetic variants, G69E, Q81P, D130N, S159N, A161S, and M162V were analyzed in the setting of a construct containing the first four CCP domains of FH responsible for C3b binding, DAA, and CA (**Figure 1**). This allowed interrogation of the functional consequences of variants in the N-terminal region of FH without the confounding presence of the C-terminal region, CCP19-20, which contains a second, stronger, C3b binding site. We have previously validated this strategy in N terminal *CFH* variants by demonstrating equivalent results in the setting of both full-length recombinant FH and the CCP1-4 recombinant FH construct used in this study (15, 27).

In keeping with the normal FH serum levels reported for these variants, all six of them were produced as folded, soluble proteins by genetically modified *Pichia pastoris*. Following purification, all FH1-4 samples were free of aggregates and degradation (**Supplementary Figure 1**).

### Effect of Variants on C3b Binding

**Figure 2:**
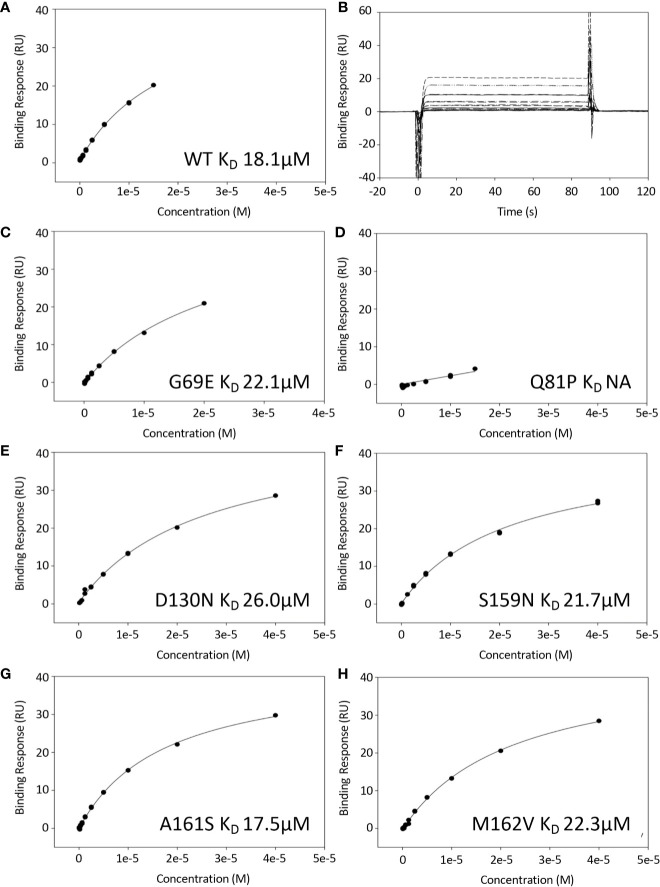
Binding of FH1-4 to C3b using surface plasmon resonance. C3b was immobilized to a CM5 sensor chip (300RU) and FH1-4 (WT and variants) were injected across (concentrations up to and including 40uM). **(B)** Sensorgrams from WT experiment demonstrating rapid association/disassociation of FH1-4 to with/from C3b. This is representative of interactions from all variants. The steady state response was plotted against concentration for all variants and shown in **(A, C–H)**. All have similar K_D_ except for Q81P where there was minimal binding at the concentrations used.

Surface plasmon resonance (SPR) was used to measure the binding interaction between FH1-4 and immobilized C3b. The plots of maximum binding response (RUs) versus concentration, used to estimate *K*_D_ values, are shown for FH1–4 WT (**Figures 2A, B**); FH1–4 G69E (**Figure 2C**); FH1–4 Q81P (**Figure 2D**); FH1–4 D130N (**Figure 2E**); FH1–4 S159N (**Figure 2F**); FH1–4 A161S (**Figure 2G**); FH1–4 M162V (**Figure 2H**).

The variant FH1-4 Q81P showed no detectable binding to C3b at the highest concentration available (40 µM Q81P in the solution flowed over the chip). All the other variants bound to C3b with a strength comparable to that of WT FH1-4. As a ratio of the *K*_D_ for C3b of WT FH1-4, *K*_D_ values were 1.2-fold for G69E, 1.4-fold for D130N, 1.2-fold for S159N, 0.97-fold for A161S and 1.2-fold for M162V (**Table 1**).

**Table 1:** Summary of the functional effects of each N-terminal *CFH* variant.

Variant	Disease	Effect of variant on				
		Affinity to C3b (Kd xWT)	Decay on SPR Assay	Decay on Sheep E (IC_50_ xWT)	Co-factor Fluid Phase	Co-factor on Sheep E (IC_50_ xWT)
G69E	AMD	1.2	↓	1.3	↔	1.1
Q81P	aHUS	↓	↓	25.1	↓	11.0
D130N	C3G, AMD	1.44	↓	2.0	↔	1.66
S159N	aHUS, AMD	1.20	↔	0.9	↔	1.54
A161S	aHUS, C3G, AMD	0.97	↔	1.3	↔	1.26
M162V	aHUS	1.23	↔	1.02	↔	1.48

### Co-Factor Activity of FH1-4 to C3b

**Figure 3:**
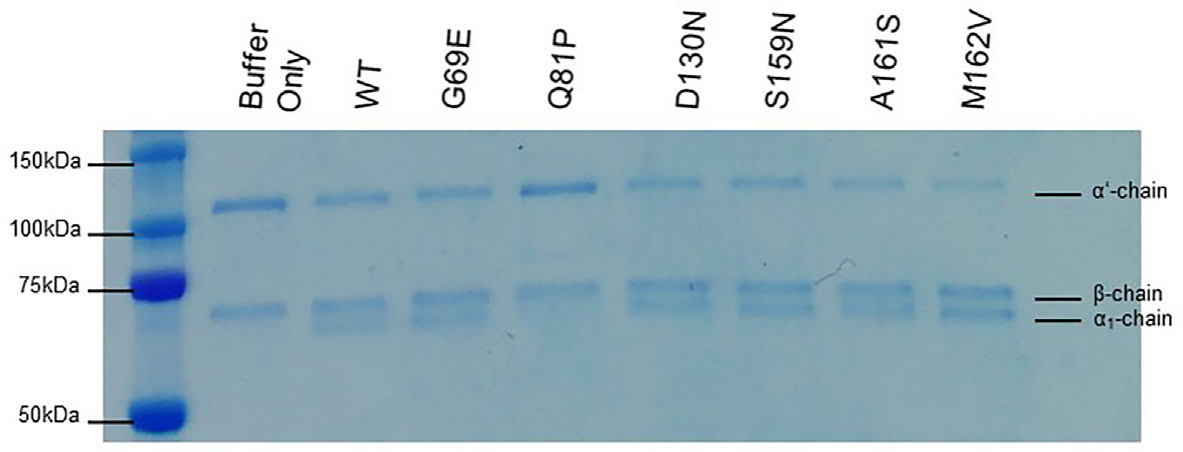
Fluid phase co-factor activity. C3b, FI, and FH1-4 were incubated for 30 min at 37°C before the addition of 2x Laemmli to stop the co-factor reaction. The products of the reaction are visualized following separation by SDS-PAGE and staining with Coomassie blue. Q81P clearly has minimal co-factor activity in the fluid phase. All other variants have detectable co-factor activity by the loss of the α’ band and the appearance of the α1 band. The β chain is the internal control. Data representative of at least 3 repeats.

To assess the effect of these sequence variations on co-factor activity, fluid-phase CA assays were initially undertaken. These demonstrated that FH1–4 Q81P has minimal CA, judging by the relative strength of the intact α’ substrate band of C3b on an SDS-polyacrylamide gel, and minimal formation of the α1 cleavage products (**Figure 3**). In these semi quantitative assays, the remaining variants demonstrated co-factor activity similar to that of WT FH1-4.

**Figure 4:**
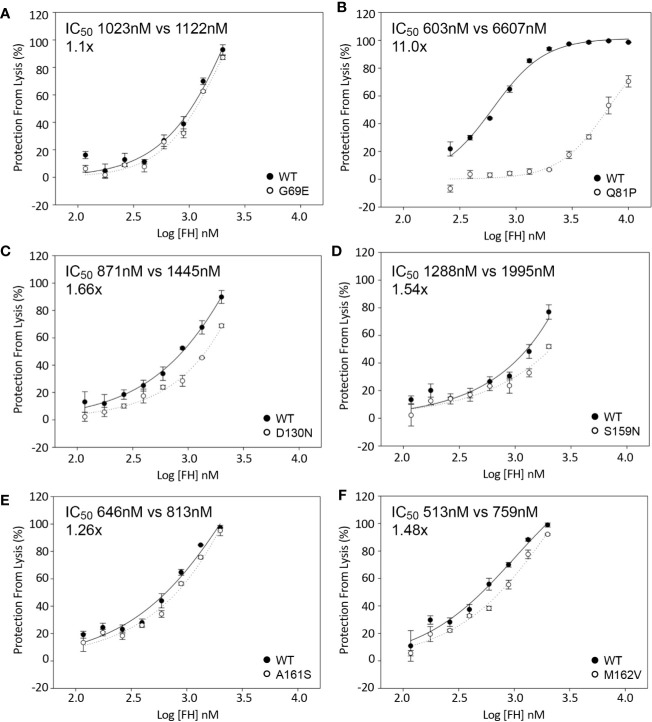
Inactivation of C3b on the surface of sheep erythrocytes. FH1-4 and FI was incubated with sheep erythrocytes with preformed surface C3b for 15 min at 25°C. Following wash steps, C3 convertase was formed on the remaining intact C3b before the instigation of lysis by the addition of ΔBHNHS. Protection from lysis was calculated as [A_410_(buffer only)−A_410_(FH)/A_410_(buffer only)*100]. **(B)** Q81P has large effect on co-factor activity compared to WT (IC_50_ 11x). **(C)** D130N demonstrated a slight loss of activity (IC_50_ 1.66x). The remaining variants demonstrated similar activity **(A, D–F)**.

The activity of each rare variant as a co-factor in the FI-mediated proteolytic cleavage of surface-bound C3b was then assessed on sheep erythrocytes. Again, Q81P FH1-4 exhibited the poorest co-factor activity (IC_50_ 11x > that of WT, **Figure 4B**). The other variants tested had surface CAs comparable to WT CA (**Figures 4A, E, F**), although D130N (IC_50_ 1.66x greater than WT) (**Figure 4C**) and S159N (IC_50_ 1.54x greater than WT) (**Figure 4D**), showed small reductions in function.

### Decay Acceleration Activity of FH1-4 to C3b

**Figure 5:**
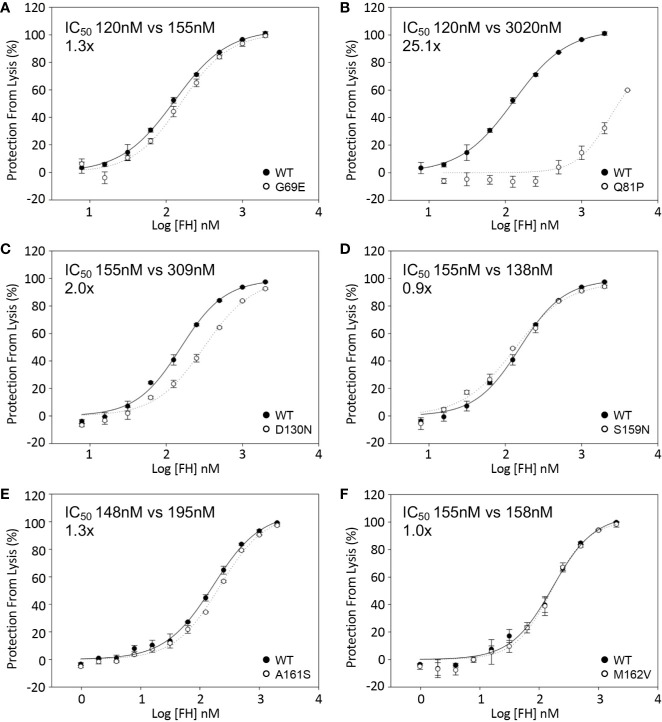
Decay acceleration of C3 convertase formed on surface of sheep erythrocytes. FB and FD were incubated with sheep erythrocytes with preformed surface C3b. The cells were then incubated with FH1-4 for 15 min at 25°C before the addition of ΔBHNHS to instigate lysis. Protection from lysis was calculated as [A_410_(buffer only)−A_410_(FH)/A_410_(buffer only)*100]. **(B)** Q81P has a profound effect of DAA with an IC_50_ 25-fold greater than wild type. The IC_50_’ s relative to WT were **(A)** 1.3x, G69E **(C)** 2x, D130N **(D)** 0.9x, S159N **(E)** 1.3, A161S **(F)** 1x, M162V.

The effect of sequence variants on the ability of FH1-4 to accelerate decay of the C3 convertase (C3bBb) assembled on the surface of sheep erythrocytes was also analyzed. These assays revealed an approximately 25-fold reduction in activity of the FH1–4 Q81P compared with FH1–4 WT (**Figure 5B**). FH-D130N displayed a small decrease in surface DAA activity (IC_50_ 2x WT) (**Figure 5C**). The remaining variants displayed still smaller differences (IC_50_: G69E 1.3x WT; S159N 0.9X WT; A161S 1.3X WT; M162V, 1.02X WT) (**Figure 5**).

**Figure 6:**
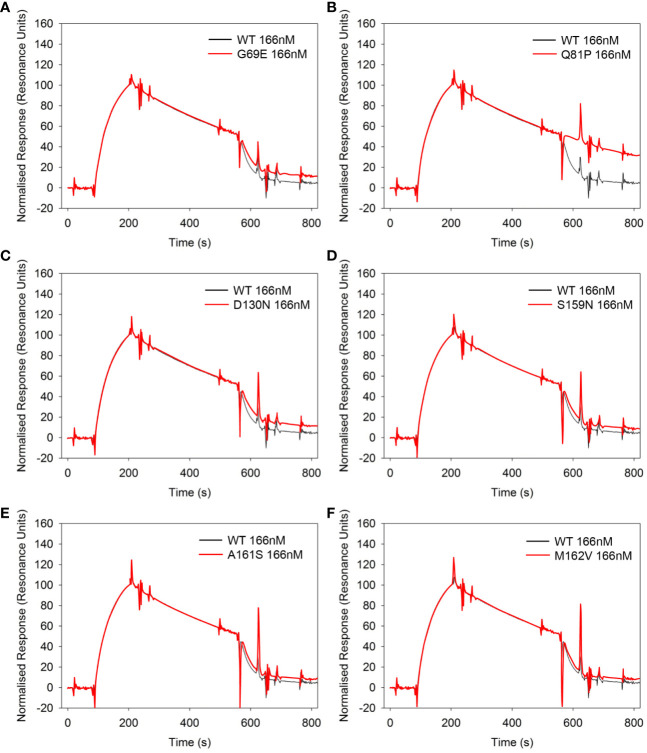
Decay acceleration of C3 convertase in real time using surface plasmon resonance. FB and FD were injected over the surface of immobilized C3b (300RU) to form a convertase as shown by an initial increase in resonance units. The convertase was allowed to decay naturally for 120s before an injection of 166nM of each FH1-4 variant **(A–F)**. All variants show the ability to accelerate decay of the AP C3 convertase apart from Q81P **(B)**. Compared to WT, G69E **(A)** and D130N **(C)** appear to be slightly less effective. Data representative of at least 2 repeats. Variant curves highlighted in red.

The effect of the six sequence variations in FH 1-4 on DAA was also analyzed in real time on an SPR chip surface (**Figures 6A–F**). In this assay the FH1–4 Q81P variant demonstrated only minimal if any effect on the rate of C3bBb decay (**Figure 6B**). Consistent with DAA measurements on erythrocytes, D130N FH1-4 had slightly less activity than WT (**Figure 6C**), as does G69E FH1-4 has less DAA than WT (**Figure 6A**).

## Discussion

The cost and speed of next-generation sequencing has now reached the point where this evolving technology can be used to alter clinical management in real time. While the results of sequencing results may be available rapidly, their interpretation can be difficult.

While the functional significance of large gene rearrangements, or frameshift mutations is clear-cut and the use of curated locus specific databases (e.g. https://www.complement-db.org/home.php) may provide data on previously described genetic variants (37), many variations in *CFH* consist of missense mutations or potential splice site changes not seen before. Interpreting these variants of unknown significance (VUSs) is particularly challenging (37).

*In silico* prediction of the consequences of amino acid changes may be attempted but in at least one *CFH* (22) case, these have proved unreliable. In the current study we provide detailed functional analysis of six rare genetic variants in the N-terminus of *CFH* that have been described in aHUS, C3G/MPGN and AMD.

The only one of these variants that had a profound effect on the complement regulatory functions of FH1-4 was Q81P, described in aHUS. It is apparent from the SPR results that its affinity for C3b is very weak compared to that of WT FH. The low affinity observed between Q81P and C3b agrees with inferences based on a co-crystal structure of C3b and CFH1–4 (36, 38) (**Figure 1**), which displays that Q81 is at the surface of the FH1-4 molecule, and is close to putative binding sites on C3b, similar to R83, a change to which (R83S) we have shown to be highly deleterious (13). In keeping with its inability to bind well to C3b, Q81P had a profound negative (25.1-fold and 11.0-fold) effect on DAA and CA, respectively. The proximal section of CCP2 and the CCP1-CCP2 linker region within FH interact through hydrophobic interactions and salt bridges to the α’ NT and MG7 domain of C3b. Q81 occupies this interface along with R78 and R83 that (when substituted by G and S respectively) also display markedly reduced affinity for C3b with consequent loss of DAA and CA (**Figure 1**).

This analysis is particularly interesting as it demonstrates that 3 variants in the same region of FH all lead to a profound defect in C3b binding with consequent abrogation of complement regulation yet cause different diseases: aHUS (R78G (15); Q81P) and C3G (R83S (13)). This suggests that the phenotype associated with these regulatory defects can be modified by genetic and environmental factors as demonstrated by Recalde et al. (39).

The G69E variation, also in CCP 1, was described in AMD. Unsurprisingly, given its location on the opposite face from the FH/C3b interface (**Figure 1**), the affinity for C3b was unaltered and there was no or only a very minor defect in CA (in the fluid phase and on cell surfaces) and there was only a subtle defect in DAA. In CCP2, D130N is predicted to be in a FH/C3b groove that allows binding of FI and subsequent cleavage of C3b, but only a small effect on co-factor activity (IC_50_ 1.66x) was observed for this variant in the sheep erythrocyte assay. Furthermore, the D130N variant produced a small but consistent effect on decay acceleration as observed on the SPR-based and cell lysis assay: such an effect on decay without loss of C3b binding has been reliably demonstrated in studies of R53H in aHUS and R53C, a variant that has been reported in association with aHUS, MPGN, C3G and AMD (16, 22, 25, 31, 32, 40) In these studies, however, there was a profound loss of DAA, suggesting the critical role of R53 in DAA. Whilst we have shown that there is an effect due to the D130N and G69E variants, the magnitude of the effect is much smaller than in causative N-terminal variants described in aHUS. This would be consistent with a minor overactivation of the AP of complement in a process that is thought to occur in more chronic disease, as observed in both of these rare variants (G69E, AMD and D130N, C3G).

Of the other variants, none displayed a detectable loss of function. Within CCP3; S159N; A161S; and M162V do sit at the interface with C3b but they displayed similar affinities for C3b as WT FH1-4 (**Figure 1**). They also displayed DAA and CA of a similar order of magnitude to WT FH1-4. The S159N and A161S variants have been demonstrated to occur in excess in AMD-case cohorts (versus control) (25) and it is possible that even the very small variations versus WT contribute to a chronic low-level complement overactivity leading to slow accumulations of complement-mediated damage over a long period of time. The minor differences that were observed were not reproducible across different assays for CA or DAA and therefore we conclude that any difference is below the detection capability of our assays.

As with previous studies *in silico* analysis here proved unreliable (22) with the profoundly perturbed Q81P variant classified as “tolerated” or a “polymorphism” in some analyses while the S159N variant (with normal function) was classified as “possibly damaging” in other analysis (**Supplementary Table 1**). As such *in silico* analysis may still be applied but should be interpreted with great caution especially when used in clinical management of disease.

In summary, we identify significant abrogation of function in an N-terminal variant of FH, Q81P, which is likely to be causative of aHUS. Two variants, G69E and D130N, demonstrated minor defects in complement regulation, which could conceivably over time lead to disease progression of more chronic diseases i.e. in C3G and AMD. Conversely, in the S159N, A161S, and M162V any functional defect was below the capacity of the experimental assays to reliably detect. This study further underlines the importance of careful biochemical assessment of disease-associated variants in complement proteins through a battery of functional assays.

## Data Availability Statment

The raw data supporting the conclusions of this article will be made available by the authors, without undue reservation.

## Author Contributions

All authors listed have made a substantial, direct, and intellectual contribution to the work and approved it for publication.

## Funding

The research was supported/funded by NIHR Newcastle Biomedical Research Centre at Newcastle upon Tyne Hospitals NHS Foundation Trust. DK was funded by Fight for Sight, the Wellcome Trust, the Medical Research Council, Kidney Research UK and Complement UK. CLH was funded by the Medical Research Council. EKSW was funded by Northern Counties Kidney Research Fund and was an MRC clinical research fellow and an NIHR Academic Clinical Lecturer. TMH is funded by Complement UK. VB is a Medical Research Council/Kidney Research UK Clinical Research Training Fellow (MR/R000913/1. PW is funded by the Wellcome trust. KSJ is a Medical Research Council (MRC) clinical research fellow (MR/R001359/1). TEC is funded by MRC Discovery Medicine North. KJM was funded by the Northern Counties Kidney Research Fund, the Newcastle Healthcare Charites and a Kidney Research UK project grant (RP7/2015).

## Conflict of Interest

EKSW has received consultancy income from Alexion Pharmaceuticals, Biocryst, and Novartis. KJM, has received consultancy income from Gemini Therapeutics Freeline Therapeutics, MPM Capital, Catalyst Biosciences. CLH has received consultancy income from Roche, GSK, Gyroscope Therapeutics, Q32 Bio, Freeline Therapeutics, Ra Pharmaceuticals, and Biocryst. DK has received consultancy income from Gyroscope Therapeutics, Alexion Pharmaceuticals, Novartis, Apellis and Sarepta.

The remaining authors declare that the research was conducted in the absence of any commercial or financial relationships that could be construed as a potential conflict of interest.
