# Supplementary material for: Functional Characterization of Rare Genetic Variants in the N-Terminus of Complement Factor H in aHUS, C3G, and AMD
Source: Front Immunol. 2021 Jan 14;11:602284. doi: 10.3389/fimmu.2020.602284 (PMC7840601; doi:10.3389/fimmu.2020.602284)

Table S1 Previously published Renal Phenotype Summary

Rare CFH variant	Q81P	D130N	S159N	A161S	M162V
Disease	Post partum aHUS	C3G	C3G	DDD	aHUS
Reference	Bruel A et al	Fremeaux-Bacchi et al 2013	Wong et al	Servais A et al	Fremeaux-Bacchi et al 2013
C3 (660 -1250 mg/l)	497	1240	690	802	N/A
C4 (90 - 380 mg/l)	326	328	200	345	N/A
CFB (90 - 320 mg/l)	59	163	N/A	108	N/A
CFH (338 - 682 mg/l)	418	663	660	602	Normal
CFI (42 to 78 mg/l)	57	55	N/A	68	N/A
Described in AMD also	-	Triebwasser et al	Triebwasser et al<br>Fritsche et al	Triebwasser et al	-

Table S2 Bioinformatic Prediction of Functional Significance

Variant		Align GVGD	SIFT	Mutation Taster	Polyphen
G69E	c.206G>A	Class C0	Tolerated (score 0.2)	Disease Causing (prob 0.538)	Probably damaging
Q81P	c.242A>C	Class C0	Tolerated (score 0.14)	Polymorphism (prob 0.998)	Probably damaging
D130N	c.388G>A	Class C0	Tolerated (score 0.68)	Polymorphism (prob 0.999)	Possibly damaging
S159N	c.476G>A	Class C0	Tolerated (score 0.35)	Polymorphism (prob 1)	Possibly damaging
A161S	c.481G>T	Class C0	Tolerated (score 0.25)	Polymorphism (prob 1)	Benign
M162V	c.484A>G	Class C0	Tolerated (score 0.21)	Polymorphism (prob 1)	Benign

Fig S1 Purified CFH1-4 variants

B

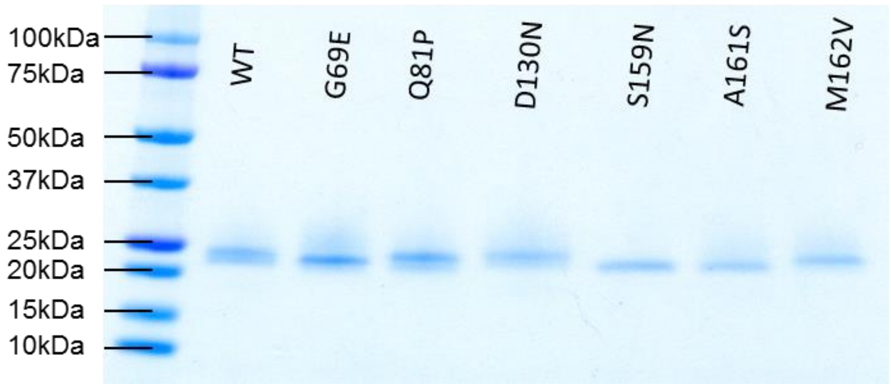

Supplement: Supplementary file 1 [file DataSheet_1.pdf]
